# Intracellular pH regulation by acid-base transporters in mammalian neurons

**DOI:** 10.3389/fphys.2014.00043

**Published:** 2014-02-13

**Authors:** Vernon A. Ruffin, Ahlam I. Salameh, Walter F. Boron, Mark D. Parker

**Affiliations:** Department of Physiology and Biophysics, Case Western Reserve UniversityOH, USA

**Keywords:** acidosis, alkalosis, brain, neurons, pH, NHE, NCBT

## Abstract

Intracellular pH (pH_i_) regulation in the brain is important in both physiological and physiopathological conditions because changes in pH_i_ generally result in altered neuronal excitability. In this review, we will cover 4 major areas: (1) The effect of pH_i_ on cellular processes in the brain, including channel activity and neuronal excitability. (2) pH_i_ homeostasis and how it is determined by the balance between rates of acid loading (*J*_L_) and extrusion (*J*_E_). The balance between *J*_E_ and *J*_L_ determine steady-state pH_i_, as well as the ability of the cell to defend pH_i_ in the face of extracellular acid-base disturbances (e.g., metabolic acidosis). (3) The properties and importance of members of the SLC4 and SLC9 families of acid-base transporters expressed in the brain that contribute to *J*_L_ (namely the Cl-HCO_3_ exchanger AE3) and *J*_E_ (the Na-H exchangers NHE1, NHE3, and NHE5 as well as the Na^+^- coupled HCO_3_^−^ transporters NBCe1, NBCn1, NDCBE, and NBCn2). (4) The effect of acid-base disturbances on neuronal function and the roles of acid-base transporters in defending neuronal pH_i_ under physiopathologic conditions.

## pH and neuronal excitability

The excitability of neurons is especially sensitive to changes in intracellular pH (pH_i_) and extracellular pH (pH_o_) due to the pH-sensitivity of intracellular and extracellular moieties on membrane proteins such as channels (Tombaugh and Somjen, 1996; Duprat et al., 1997; Waldmann et al., 1997; Ruffin et al., 2008), transporters (Irwin et al., 1994; Park et al., 2010; Adijanto and Philp, 2012), receptors (Giffard et al., 1990; Tang et al., 1990; Traynelis and Cull-Candy, 1990; McDonald et al., 1998), and ATPase pumps (Pick and Karlish, 1982; Wolosker et al., 1997). Together these proteins (1) govern the resting membrane potential of neurons, (2) affect neuronal responsiveness to agonists and antagonists, (3) set the threshold for firing an action potential, (4) influence the duration/amplitude of the action potential, (5) determine the length of the refractory period, and (6) synchronize neuronal network activity. These properties endow neurons with the ability to communicate with other neurons and glial cells within the nervous system (for functions such as learning, behavior, conscious thought, and unconscious homeostatic regulation), and with cells outside the nervous system (for functions such as motor control and endocrine regulation). Table 1 list examples of pH sensitive proteins and activities involved in setting neuronal excitability. The relationship between pH and neuronal excitability has been extensively reviewed by others (Balestrino and Somjen, 1988; Church, 1992; Tombaugh and Somjen, 1996; Dean et al., 2001; Makani and Chesler, 2007; Pavlov et al., 2013). Note that the link between pH and neuronal excitability is not a simple one: some neurons (e.g., chemosensitive neurons) exhibit enhanced excitability in response to an acid-load, whereas others (e.g., hippocampal neurons) may exhibit reduced excitability. The direction of the response presumably depends on the pH-responsiveness of the individual channels, transporters, and receptors that are responsible for dictating overall excitability in each neuron.

**Table 1:** **Examples of pH-sensitive membrane proteins expressed in neurons**.

**Protein class**	**Example protein(s)**	**Acidosis effect**	**Reference**
Ion channels	Inward rectifier K^+^ channel, HIR (Kir2.3)	Decreases single channel conductance	Coulter et al., 1995
	Two-pore domain K^+^ channel, TASK	Reduces current	Duprat et al., 1997; Tombaugh and Somjen, 1997
	Voltage-gated Na^+^, K^+,^ and Ca^2+^ channels	Influences numerous conductance and gating properties	Tombaugh and Somjen, 1996
	Na^+^-activated K^+^ channel, K_Na_	Reduces activity	Ruffin et al., 2008
	Acid-sensing channel, ASIC	Increases activity	Waldmann et al., 1997
Receptors	NMDA receptor	Reduces current	Giffard et al., 1990
	AMPA receptor	Reduces current	McDonald et al., 1998
Transporters	Electroneutral Na/HCO_3_ cotransporter, NBCn1	Increases expression	Park et al., 2010
	Monocarboxylate transporters	Increases activity	Manning Fox et al., 2000; Adijanto and Philp, 2012; Halestrap, 2012
Pumps	Ca^2+^ ATPase	Increases activity	Pick and Karlish, 1982; Irwin et al., 1994; OuYang et al., 1994; Wolosker et al., 1997

Glial cells are not excitable cells and experience only small changes in membrane potential compared to neurons. Although glial cells are not the focus of this review, it is important to address their critical role in optimal brain function. Traditionally, glial cells have been called neural supportive cells because they produce growth factors and recycle neurotransmitters (astrocytes), assist in rapid electrical transmission (oligodendrocytes), and scavenge compromised cells in addition to cellular debris (microglia). Glia, together with choroid plexus epithelia (Schmitt et al., 2000; Damkier et al., 2010a; Christensen et al., 2013), control the composition—including the pH—of the extracellular cerebrospinal fluid (CSF) that bathes neurons (Chesler and Kraig, 1989; Deitmer and Rose, 1996; Bevensee et al., 1997; Brookes, 1997; Chesler, 2003; Ro and Carson, 2004; Ekdahl et al., 2009).

Models of seizure indicate that the acidification that follows intense firing (Chesler and Kaila, 1992; Jacobs et al., 2008), a phenomenon that likely forms part of a mechanism that prevents excessive firing by dampening neuronal excitability (Hormuzdi et al., 2004), is a major challenge to neuronal pH. Intriguingly, the main clinical presentations of several neurodegenerative disease states include signs of decreased brain pH. Examples include Alzheimer's disease (Demetrius and Simon, 2012), Parkinson's disease (Mattson et al., 1999), and multiple sclerosis (Vergo et al., 2011). Even disease states originating outside of the brain (e.g., metabolic acidosis) can affect the pH of the brain. Given the link between pH and neuronal function, it is probable that such alterations in pH compromise brain function, contributing to the neurological symptoms of these diseases.

The regulation of cytosolic pH in most cells, including neurons, is an active process, since H^+^ ions are not passively distributed across the cell membrane (Roos and Boron, 1981). In this brief review, we will provide an overview of the nature, function, and importance of the major acid-loading and acid-extruding proteins that contribute to neuronal pH homeostasis. We will also consider the pathologies that are associated with defective neuronal acid-base homeostasis and how the homeostatic systems respond in the face of pathological acid-base disturbances.

## Neuronal pH homeostasis

**Figure 1:**
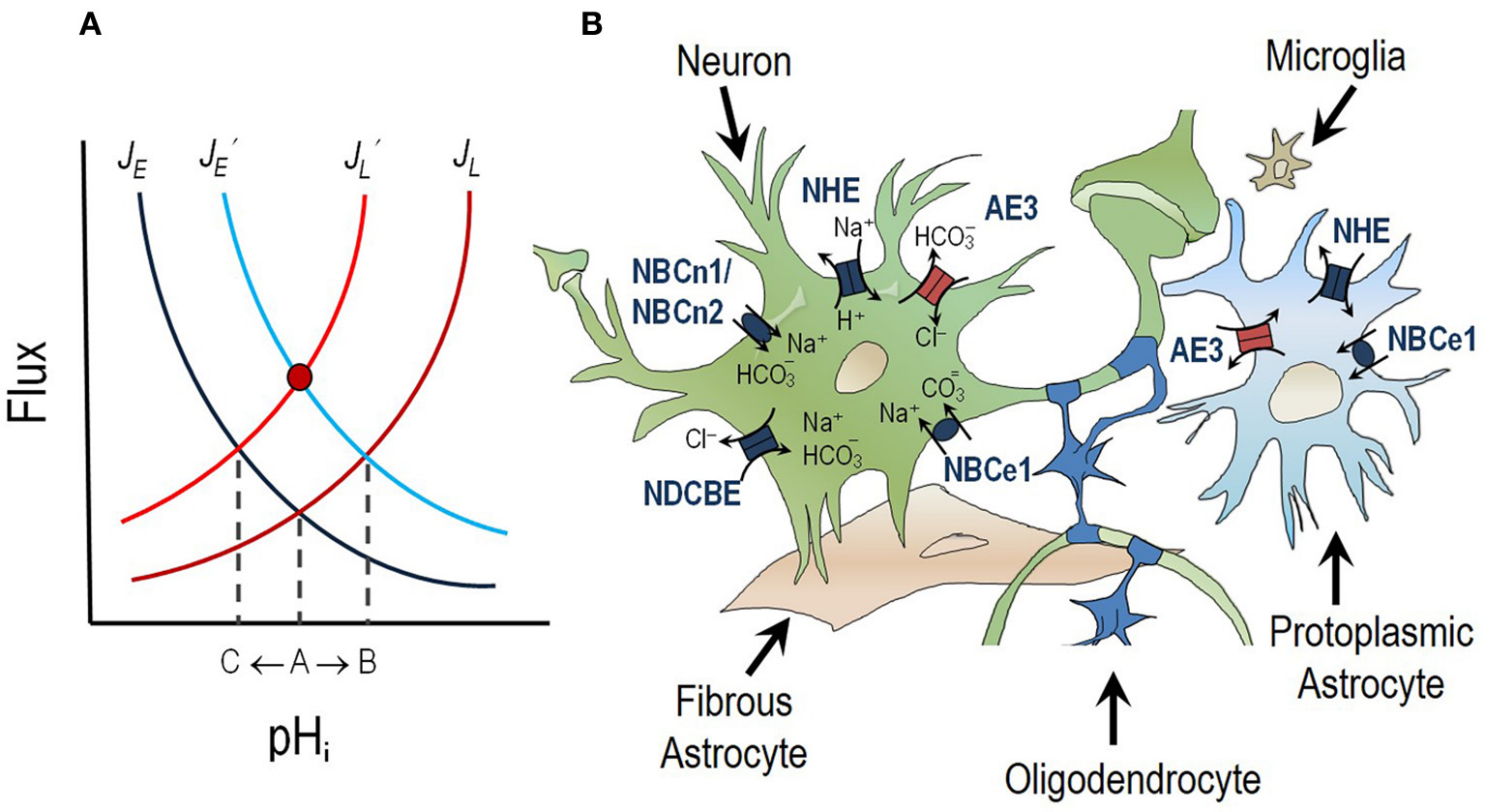
**pH regulation in the central nervous system. (A)** Steady-state pH_i_ is dependent on the balance between the rate of acid extrusion (*J*_E_) and the rate of acid loading (*J*_L_). Steady-state pH_i_ is achieved when *J*_E_ = *J*_L_ (intersection of dark-blue and dark-red lines: point A). If *J*_E_ increases (*J*′_E_) the steady-state pH_i_ will shift to a more alkaline value (intersection of light-blue and dark-red lines: point B). If *J*_L_ increases (*J*′_L_) the steady-state pH will shift to a more acidic value (intersection of dark-blue and light-red lines: point C). If the rise in *J*_E_ is matched by an equal increase in *J*_L_ (*J*′_E_ = *J*′_L_) there will be no net change in pH_i_ (red circle). This is known as a compensatory response. **(B)** Neurons, astrocytes and oligodendrocytes express two classes of acid-base transporting proteins; acid loaders (red) and acid extruders (dark blue).

Pioneering work in invertebrate models first identified the importance of neuronal pH regulation (Boron and De Weer, 1976a,b; Thomas, 1976). In vertebrates, extensive research has been performed on pH regulation in hippocampal neurons. The typical resting or “steady-state” pH_i_ of a hippocampal neuron in CO_2_/HCO_3_^−^-containing media is ~7.03–7.46. depending on the preparation, whereas the pH_o_ is ~7.35 (Raley-Susman et al., 1991, 1993; Schwiening and Boron, 1994; Baxter and Church, 1996; Bevensee et al., 1996; Church et al., 1998; Smith et al., 1998; Vincent et al., 1999). Steady-state pH_i_ is dependent on the balance between the rate of acid loading (*J*_L_, i.e., rate of acid influx/generation or alkali efflux/consumption) and the rate of acid extrusion (*J*_E_, i.e., rate of acid efflux/consumption or alkali influx/generation). Steady state pH_i_ is achieved when *J*_E_ = *J*_L_ (intersection of red and blue lines in Figure 1A). It is important to note that, at steady-state, the opposing acid-loading and acid-extruding processes are not stopped but are proceeding at equal rates, thus their combined action results in no pH change.

Acid loading in neurons—a process that tends to lower pH_i_—predominantly results from the accumulation of metabolically generated H^+^ (such as that produced by aerobic or anaerobic metabolism during neuronal firing: Chesler, 2003) and the extrusion of HCO_3_^−^ from cells via a Cl-HCO_3_ (anion) exchanger of the SLC4 solute carrier family (see section titled The Chloride-Bicarbonate Exchanger AE3 and Figure 1B). Acid-loading processes tend to restore steady-state pH_i_ after an alkali load.

Acid extrusion—a process that tends to raise pH_i_—in neurons is typically achieved by the action of the SLC4 and SLC9 families of Na^+^-coupled transport proteins. Both transporter families that take advantage of the inwardly directed Na^+^ gradient established by the Na^+^, K^+^ ATPase to either extrude H^+^ from the cell (in the case of Na-H exchangers (NHEs), see section titled Sodium-Hydrogen Exchangers NHE1, NHE3, and NHE5 and Figure 1B) or to accumulate a weak base, such as HCO_3_^−^ (in the case of Na/HCO_3_ cotransporters, see section titled Sodium-coupled Bicarbonate Transporters and Figure 1B). Acid-extruding processes tend to restore steady-state pH_i_ after an acid load, such as that which might result from intense neuronal activity (Chesler and Kraig, 1989; Kaila et al., 1991).

Of course, transmembrane acid-loading processes tend to alkalinize the cell surface (raise pH_s_) and transmembrane acid-extruding processes tend to acidify the cell surface (lower pH_s_), thereby also potentially impacting the function of membrane proteins in the brain with extracellular pH-sensitive moieties. In practice, extracellular membrane-associated carbonic anhydrases catalyze the interconversion of H^+^ + HCO_3_^−^ ↔ CO_2_ + H_2_O at the cell surface, which would tend to promote dissipation of pH gradients. A consideration of the CO_2_/HCO_3_^−^ buffering system and whole-body pH homeostasis is provided elsewhere (Giebisch and Windhager, 2009; Boron, 2012; Bevensee and Boron, 2013).

## Neuronal acid-base transporters

### Acid loaders

#### The chloride-bicarbonate exchanger AE3

***Molecular identity***. The main acid loader that is predicted to contribute to *J*_L_ in neurons is the Cl-HCO_3_ exchanger AE3 (Anion Exchanger 3, encoded by the *SLC4A3* gene). AE3 was the third member of the 10 members of the SLC4 solute carrier family to be cloned and characterized and, like its close relatives AE1 and AE2 (Alper, 2009), mediates the stilbene-sensitive, electroneutral exchange of one Cl^−^ for one HCO_3_^−^ (Sterling and Casey, 1999). As we will see later, not all SLC4 members are acid loaders: indeed the majority are acid extruders (see Na^+^-coupled HCO_3_^−^ transporters, SLC4A4-8 in Section titled Sodium-coupled Bicarbonate Transporters). In mammals SLC4A3 encodes two alternative gene products: bAE3 (abundant in the brain, often referred to as AE3fl, full-length) and cAE3 (abundant in cardiac tissue). bAE3 is expressed from a different promoter than cAE3 and includes a longer and different Nt appendage. Artificial truncation of the bAE3-specific sequence appears to confer a lesser functional expression on the transporter, consistent with the hypothesis that this sequence is autostimulatory. The appendage has also been reported to include two SH3 domains and a PKC site indicating a possible role for modulation by extrinsic signals (Sterling and Casey, 1999; Alvarez et al., 2001).

***Distribution***. bAE3 transcripts and protein are expressed throughout the central nervous system. In mice, bAE3 transcripts are especially abundant in pyramidal neurons of the hippocampal (HC) formation (Kopito et al., 1989; Hentschke et al., 2006) although western blotting of brain regions shows AE3 protein to be similarly abundant in the cerebral cortex (CX), cerebellum (CB), and brainstem-diencephalon (BD): (Xue et al., 2003). Western blotting of fractionated cells suggests that AE3 protein expression in the brain is mainly in neurons rather than in astrocytes (Hentschke et al., 2006). However, in the retina of rats, bAE3 is located in the basal end feet of Müller cells (glia) and it is cAE3 that is expressed in horizontal cells (neurons): (Kobayashi et al., 1994; Alvarez et al., 2007).

***Influence on neuronal pH_i_***. Cl-HCO_3_ exchange is a feature of adult neurons and AE3 is the sole Cl-HCO_3_ exchanger in neurons as evidenced by the absence of Cl-HCO_3_ exchange activity in AE3-null mice (Hentschke et al., 2006). Interestingly, despite evidence for the presence of AE3 transcripts in embryonic mouse and rat brain, (Hentschke et al., 2006) neurons from fetal mice exhibit no substantial Cl-HCO_3_ exchange activity as if AE3 protein is absent or otherwise indisposed (Raley-Susman et al., 1993; Vilas et al., 2009). However, as mentioned below, AE3-like activity is evident as a damping mechanism when acid-extruders are in robust operation. Due to the prevailing ion gradients and probably also due to the relative substrate affinities of the intracellular and extracellular ion translocation sites, AE3 typically extrudes HCO_3_^−^ in exchange for extracellular Cl^−^, thereby tending to lower pH_i_ (raise pH_s_) and raise [Cl^−^]_i_. Evidence for the role of AE3 as an acid loader is provided by the observations that (1) COS cells expressing AE3 exhibit a markedly lower pH_i_ than control cells that do not express AE3 (Kopito et al., 1989) and (2) hippocampal neurons from embryonic mice exhibit an enhanced rate of acid-extrusion in the presence of DIDS or in the absence of extracellular Cl^−^: both maneuvers that would block AE3 action (Svichar et al., 2009). Furthermore, HC neurons from adult Ae3-null mice exhibit a slightly higher pH_i_ than wild-type neurons, although the authors of the study note that the pH difference did not achieve statistical significance in their study (Hentschke et al., 2006). A Cl-HCO_3_ exchanger, likely AE3, also contributes to *J*_L_ in non-chemosensitive and some chemosensitive neurons of the medulla oblongata (Ritucci et al., 1998; Meier et al., 2007).

***Importance for neuronal function***. Mice lacking acid-extruders of the SLC4 family tend to exhibit evidence of reduced neuronal excitability (see section titled Sodium-coupled Bicarbonate Transporters), thus it seems fitting that a missense mutation in AE3, an acid-loader, is associated with idiopathic generalized epilepsy (Sander et al., 2002). Subsequent work has shown that the mutant AE3 is functionally defective in a heterologous system (Vilas et al., 2009). Moreover, a strain of Ae3-null mouse exhibits lower seizure threshold in response to proconvulsants and a greater seizure-mortality consistent with enhanced neuronal excitability (Hentschke et al., 2006). Ae3-null mice exhibit a reduced respiratory rate consistent with a contribution to the resting pH in chemosensitive neurons that, unlike other neurons, are stimulated by lowered pH_i_ (Meier et al., 2007). Finally, in the mouse retina, the importance of AE3 for maintaining appropriate electrical excitability is indicated by the association of AE3-deficiency with blindness (Alvarez et al., 2007). However, it is not clear if any of these indicators of altered neuronal excitability are entirely due to defective pH regulation, or if they are related to altered Cl^−^ accumulation, another factor known to influence neuronal excitability (Irie et al., 1998; Kahle et al., 2005).

### Acid extruders

In neurons and astrocytes, the main acid extruders contributing to *J*_E_ are the NHEs in the SLC9 family of solute carriers (recently reviewed by Donowitz et al., 2013) and the Na^+^-coupled HCO_3_^−^ transporters (NCBTs) in the SLC4 family (recently reviewed by Parker and Boron, 2013).

#### Sodium-hydrogen exchangers NHE1, NHE3, and NHE5

**Figure 2:**
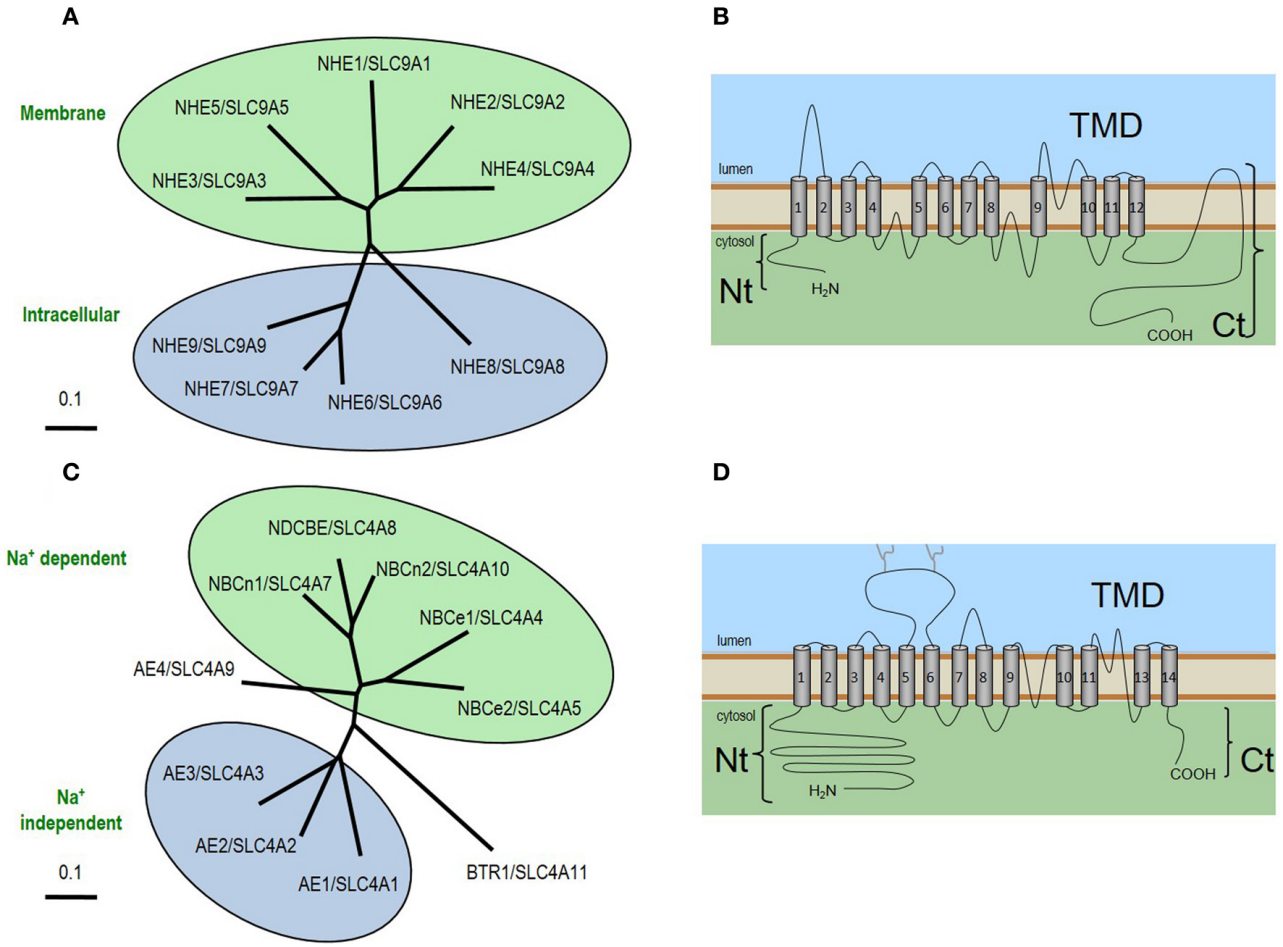
**Neuronal acid-base transporters of the SLC9 and SLC4 families. (A)** The relatedness of SLC9 family proteins. **(B)** The general topology of SLC9 proteins. The human SLC9 gene family of solute carriers consists of 9 members which encode proteins containing a ~450 aa N-terminus composed of 12 membrane spans that form the transmembrane domain (TMD) where the exchange of extracellular Na^+^ and intracellular H^+^ occurs, and a C-terminus of varying length (~125–440 aa) depending on isoform, that is involved in the regulation of exchange activity. **(C)** The relatedness of SLC4 family proteins. **(D)** The general topology of SLC4 proteins. The extended N-terminus and C-terminus are linked via a TMD that includes 14 transmembrane spans, one of which (transmembrane span 12) is believed to be an extended non-helical region. Each NCBT gene encodes multiple products that differ from each other in their extreme Nt or Ct sequence (Boron et al., 2009).

***Molecular identity***. The main HCO_3_-independent acid loaders that are predicted to contribute to *J*_E_ in neurons and astrocytes are the NHEs (Orlowski and Grinstein, 2004; Donowitz et al., 2013). NHE1 (neurons and astrocytes), NHE3 (chemosensitive neurons), and NHE5 (neurons) are encoded by the *SLC9A1, SLC9A3*, and *SLC9A5* genes, respectively. Three of five members of the SLC9 family mediate the electroneutral exchange of one Na^+^ for one H^+^ across the plasma membrane. The other four other members, NHE6-NHE9, are intracellular K-H exchangers. The general topology and relatedness of family members are show in Figures 2A,B. SLC9 family members have a short Nt and an extensive Ct that plays a regulatory role (Orlowski and Grinstein, 2004; Donowitz et al., 2013). NHE1 is far more sensitive to amiloride derivatives than either NHE3 or NHE5 (Counillon et al., 1993; Orlowski, 1993).

***Distribution***. NHE1 exhibits the broadest distribution of all the NHE isoforms throughout the body and has been identified in multiple brain regions (Ma and Haddad, 1997; Kanaan et al., 2007), both in neurons (e.g., cultured mouse HC and neocortical neurons: Sin et al., 2009; Diering et al., 2011) and astrocytes (e.g., cultured rat HC astrocytes: Pizzonia et al., 1996). NHE3 exhibits robust expression in cerebellar Purkinje cells (Ma and Haddad, 1997) and also in chemosensitive, ventrolateral neurons in the brainstem/medulla oblongata (Wiemann et al., 1999, 2005; Kiwull-Schöne et al., 2001, 2007). NHE5 expression is predominantly detected in the brain (Klanke et al., 1995; Attaphitaya et al., 1999) with robust expression in multiple brain regions (Attaphitaya et al., 1999; Baird et al., 1999). At the subcellular level, NHE5 protein has been detected in the synapses of HC pyramidal neurons of mice in both a subset of inhibitory and excitable synapses (Diering et al., 2011).

***Influence on neuronal pH_i_***. Due to the prevailing ion gradients, NHEs typically extrude H^+^ in exchange for extracellular Na^+^ (taking advantage of the inwardly directed gradient for Na^+^), thereby tending to raise pH_i_ (e.g., while restoring steady-state pH_i_ after an acid load) and lower pH_s_. Evidence for the role of NHE1 as an acid extruder is provided by the observation that CA1 neurons from Nhe1-null mice exhibit a significantly lower steady-state pH_i_ than wild-type neurons (7.17 vs 7.25) and a slower recovery from NH_4_-induced acid-loads: with some individual neurons being completely unable to recover from the acid-load in the absence of HCO_3_^−^ (Yao et al., 1999). NHE1 is also a major contributor to pH_i_ recovery in chemosensitive neurons of the retrotrapezoid nucleus (RTN) and nucleus tractus solitarius (NTS) (Kersh et al., 2009). In addition to the direct effects of NHE1 absence, loss of NHE1 also has indirect effects on the mechanism of neuronal pH_i_ regulation due to the compensatory downregulation of AE3 in the HC and upregulation of NHE3 (in the CB) and NBCe1 (in the BD), all of which would tend to compensate for the loss of NHE1-mediated *J*_E_ (Xue et al., 2003).

The importance of NHE3 to pH regulation in chemosensitive neurons is indicated by the following studies: (1) inhibition of NHE3 lowers the steady-state pH_i_ of medullary raphé chemosensitive neurons by 0.1 unit (Wiemann et al., 1999) and (2) pharmacological data suggests that NHE3 contributes, along with NHE1, to *J*_E_ in RTN neurons (Kersh et al., 2009).

One study of cultured HC neurons revealed that NHE5 contributes to acid-extrusion in the dendritic spine during enhanced neuronal activity (effected by NMDA receptor activation), a process that would tend to acidify the synaptic cleft (pH_s_): (Diering et al., 2011).

***Importance for neuronal function***. A spontaneous slow wave epilepsy was mapped to a null-mutation in the *Nhe1* gene locus in one strain of mouse, (Cox et al., 1997) a finding that was bolstered by the exhibition by targeted-null mice of ataxia, apparent absence-seizures, and a post-mortem appearance consistent with seizure-related death (Bell et al., 1999). Indeed, CA1 neurons from spontaneously Nhe1-null mice are demonstrated to have enhanced excitability compared to wild-type neurons (Gu et al., 2001; Xia et al., 2003). However, the underlying cause is complex because other changes are detected in these neurons that would tend to enhance excitability, such as increased Na^+^ channel density (Gu et al., 2001; Xia et al., 2003), and a reduction of delta-opioid receptor expression (Zhao et al., 2005).

Chemosensitive neurons are unusually stimulated by acidification, a phenomenon that serves to stimulate exhalation of CO_2_ (a potential acid) during acidosis. Three lines of evidence suggest that NHE3 plays a role in maintaining steady-state pH_i_ in these cells, thereby setting resting ventilation rate: (1) in rats, NHE3 blockade results in a lowering of pH_i_, causing a great increase in bioelectric activity (Wiemann et al., 1999; Kiwull-Schöne et al., 2001), (2) systemic application of an NHE3 blocker to rats causes an increased respiratory frequency (Ribas-Salgueiro et al., 2009), and (3) rabbits with lower NHE3 mRNA abundance tend to exhibit greater ventilation rates than these with a higher NHE3 mRNA abundance (Wiemann et al., 2005).

It has been suggested that NHE5 could play a critical role in the synaptic pH regulation during the firing of action potentials. In cultured hippocampal neurons, the activation of NMDA receptors recruits NHE5 protein to the membrane surface where it not only fosters focal-synaptic-cleft acidification, but also suppresses activity-induced dendritic spine growth by an autocrine feedback mechanism (Diering et al., 2011). Accordingly, knockdown of NHE5 or overexpression of a dominant-negative mutant of NHE5 in cultured hippocampal neurons causes dendritic spine overgrowth (Diering et al., 2011).

#### Sodium-coupled bicarbonate transporters

***Molecular identity***. The main HCO_3_-dependent acid loaders that are predicted to contribute to *J*_E_ in neurons and astrocytes are the Na^+^-coupled HCO_3_ transporters (NCBTs): NBCe1, NBCn1, NDCBE, and NBCn2. NCBTs—like the acid loader AE3— are members of the SLC4 solute carrier family (Figure 2C) and share the same general topology (Figure 2D). However, unlike AE3, and more like NHEs, the common molecular action of NCBTs takes advantage of the inwardly directed Na^+^ gradient to promote the influx of HCO_3_^−^, thereby tending to raise pH_i_. NBCe1 (encoded by *SLC4A4*) is an electrogenic Na/HCO_3_ cotransporter that mediates the coupled influx of 1 Na^+^ plus 2 HCO_3_^−^ equivalents (Romero et al., 1997). NBCn1 (encoded by *SLC4A7*) is an electroneutral NCBT that mediates the coupled influx of 1 Na^+^ and 1 HCO_3_^−^ equivalent; NBCn1 is unique among the NCBTs inasmuch as it exhibits a pronounced HCO_3_^−^-independent Na^+^ flux (Choi et al., 2000). NDCBE (*SLC4A8*) is a Na^+^-driven Cl-HCO_3_ exchanger that mediates the electroneutral exchange of 1 Na^+^ plus 2 HCO_3_^−^ equivalents for 1 Cl^−^(Grichtchenko et al., 2001). NBCn2/NCBE (*SLC4A10*) has a controversial molecular action in as much it is appears capable of NDCBE-like activity in under certain assay conditions (Wang et al., 2000; Parker et al., 2008; Damkier et al., 2010b) yet the protein expressed in *Xenopus* oocytes mediates NBCn1-like electroneutral Na^+^ and HCO_3_^−^ cotransport alongside futile Cl-Cl exchange cycles that result in no net movement of Cl^−^(Parker et al., 2008). The majority of SLC4 members are inhibited by disulfonic stilbene derivatives such as DIDS, although NBCn1 is relatively poorly inhibited by DIDS (Choi et al., 2000).

***Distribution***. NBCe1 transcripts and protein are expressed throughout the central nervous system in both neurons and astrocytes (Majumdar et al., 2008). The three electroneutral NCBTs (NBCn1, NDCBE, and NBCn2) are also expressed throughout the brain—in fact the brain is the major site of expression for NDCBE and NBCn2 (Grichtchenko et al., 2001; Parker et al., 2008)—and are especially abundant in HC neurons (Damkier et al., 2007; Boedtkjer et al., 2008; Chen et al., 2008a,b; Jacobs et al., 2008; Cooper et al., 2009). All chemosensitive neurons from the medullary raphe appear to express all NCBTs in culture (Coley et al., 2013). *In situ*, NBCn1 expression has been detected in GABAergic and non-GABAergic HC neurons (Cooper et al., 2005) as well as in the post-synaptic dendritic spines of embryonic rat neurons (Cooper et al., 2005; Park et al., 2010). In addition, an NBCn1-like activity is present in locus coeruleus neurons (Kersh et al., 2009). NDCBE expression has been detected in presynaptic nerve endings of glutamatergic neurons, with a lesser presence in GABAergic neurons (Sinning et al., 2011; Burette et al., 2012). NBCn2 expression has been detected in pre- and post-synaptic compartments of GABAergic HC neurons (Sinning and Hübner, 2013).

***Influence on neuronal pH_i_***. It has long been recognized that the pH_i_ and the excitability of HC neurons is enhanced in the presence vs. the absence of CO_2_/HCO_3_ (Yao et al., 1999) and also that neuronal *J*_E_ is enhanced in the presence of CO_2_/HCO_3_ (Bevensee et al., 1996). Being expressed in neurons, all four of the NCBTs mentioned the in the previous section likely contribute to *J*_E_. Neurons from mice lacking NBCe1 (Svichar et al., 2011) and brain slices from mice lacking NBCn2 (Jacobs et al., 2008) exhibit substantial deficits in *J*_E_. However, we are not aware of any studies that directly address the role of NBCn1 or NDCBE in setting steady-state pH_i_, or contributing to *J*_E_.

***Importance for neuronal function***. Individuals with mutations in NBCe1 often exhibit intellectual impairments that could be a result of dysfunctional neuronal pH_i_ regulation (Igarashi et al., 2001, 2002; Horita et al., 2005; Demirci et al., 2006). However, these individuals also exhibit a severe metabolic acidosis (NBCe1 is required in the kidney maintain plasma HCO_3_^−^) that could itself disturb brain pH (see section titled Metabolic Acidosis).

Mice lacking NDCBE (Sinning et al., 2011) and NBCn2 (Jacobs et al., 2008) exhibit greater resistance to seizure-induction consistent with the hypothesis that these transporters are normally required to contribute to *J*_E_ and maintain neuronal excitability. Furthermore, in humans, the *SLC4A10* (NBCn2) gene-locus is linked with epilepsy and autism (Sebat et al., 2007; Gurnett et al., 2008; Krepischi et al., 2010). However, as mentioned above in relation to AE3-null mice, both transporters are capable of influencing Cl^−^ accumulation which may itself impact excitability. Another factor that cannot be ignored is that NBCn2 is expressed in the choroid plexus where it is a key player in a pathway that controls [HCO_3_^−^] in the CSF (Jacobs et al., 2008), thus the effect of NBCn2 loss on neuronal excitability could have an indirect component. One other NCBT that we have not considered above is NBCe2, the second electrogenic NCBT, which is encoded by *SLC4A5* (Sassani et al., 2002; Virkki et al., 2002). NBCe2 is another key player in CSF secretion by the choroid plexus (Bouzinova et al., 2005; Millar and Brown, 2008), a factor that likely underlies the reduced neuronal excitability evident in brain slices from Nbce2-null mice (Kao et al., 2011); expression of NBCe2 has not been demonstrated in neurons.

### Other plasma membrane proteins that influence neuronal pH

Although this review focuses on the major pH regulating protein in the brain, other factors contribute either directly or indirectly to pH_i_, pH_s_, and pH_o_ through the influx or efflux of acid-base equivalents. For instance, the H^+^-coupled monocarboxylate transporters (MCT1-4) play major role in transporting carboxylic acids, e.g., lactate and pyruvate, between neurons and astrocytes which are then used as a source of energy (Adijanto and Philp, 2012; Choi et al., 2012). The activation of NMDA receptors induces a Ca^2+^ dependent pH_i_ acidification in rat HC neurons (Irwin et al., 1994) that is likely due to Ca-H exchange mediated by the Ca^2+^-ATPase (Makani and Chesler, 2010). The extent of the drop in neuronal pH_i_ due to Ca^2+^-ATPase action during electrical activity is limited by a depolarization-induced alkalization that is likely mediated by the proton-efflux channel H_V_1(Meech and Thomas, 1987; Cheng et al., 2008; Meech, 2012). Finally, we must not discount the contribution of acid-base transporters in the astrocytes (Chesler, 2003) and choroid plexus epithelia (Damkier et al., 2010a, 2013), cells that control the composition of the brain extracellular fluid and thus indirectly influence the pH of neurons.

## Physiopathological acid-base disturbances

The four major acid base disturbances in the body are respiratory acidosis, respiratory alkalosis, metabolic acidosis, and metabolic alkalosis. In the following section we will discuss (1) the cause of each of these disturbances, (2) the clinical presentation of these disturbances, (3) the effect of these disturbances on pH_i_, (4) the effect of these pH changes on the acid base transporter activity, and (5) the way the body compensates for these disturbances using the respiratory and renal systems.

### Respiratory acidosis (hypercapnia)

Respiratory acidosis results from inability to eliminate, from the body, the CO_2_ that is produced from cellular respiration. As a consequence the partial pressure of CO_2_ (pCO_2_) in the blood rises and the pH of the blood decreases as described by the Henderson-Hasselbalch equation (Hills, 1973; Hurn et al., 1991). Some of the causes of respiratory acidosis are CNS depression, neuromuscular disease, chronic obstructive pulmonary disease (COPD), sleep apnea, alveolar hypoventilation, ischemia, lung disease, and obesity. Clinical symptoms of hypercapnia include anxiety, pulmonary hypertension, tachypnea, extrasystoles, muscle twitches, and reduced neural activity. Prolonged hypercapnia results in disorientation, convulsions, unconsciousness, and eventually death (Ayers and Warrington, 2008).

Specialized neuronal groups (respiratory chemoreceptors) within the brain protect against the compromised neuronal function associated with ECF (extracellular fluid) acidosis. Respiratory chemoreceptors were first identified on the surface of the medulla (Mitchell et al., 1963; Loeschcke et al., 1970; Schlaefke et al., 1970), and later throughout the brainstem and hypothalamus (Berquin et al., 2000). Respiratory chemoreceptors chemically sense increased CO_2_ or H^+^ production. In response to the stimulus of lowered pH_i_ that accompanies this increased CO_2_ or H^+^ production, the excitability of these cells is enhanced, which increases respiratory drive, thereby appropriately adjusting the pH of the ECF (blood, CSF, and interstitial fluid): (Douglas et al., 2001; Putnam, 2001; Bouyer et al., 2004; Richerson, 2004; Hodges and Richerson, 2010).

Longer term exposure to elevated pCO_2_ increases the expression of acid extruders (NHE1 and NBCn1) and decreases the expression of the acid loader AE3 throughout the mouse brain, mostly prominently in the cortex, and especially in neonates (Kanaan et al., 2007). This may reflect a compensatory mechanism that would counter acidosis and tend to maintain neuronal excitability. Recent data points to a potential genetic link between NHE3, breathing control, and sudden infant death syndrome (Wiemann et al., 2005, 2008; Poetsch et al., 2010).

### Respiratory alkalosis (hypocapnia)

Respiratory alkalosis results from the excess elimination of CO_2_ from the body. As a result, pCO_2_ decreases and the pH of the blood increases. Some of the causes of respiratory alkalosis are hypoxia, hyperventilation, CNS disorders (meningitis), and drugs. The decrease in CO_2_ is usually well tolerated, although there are typical clinical symptoms that include confusion, dizziness, muscle cramps, chest wall tightness, and tetany in the extremities (Ayers and Warrington, 2008).

The main stimulus for ventilation is increased CO_2_, and as a result the decreased pCO_2_ present in respiratory alkalosis can suppress breathing. As breathing decreases, the pCO_2_ rises and returns the blood pH to lower values. The decreased breathing also results in decreased O_2_ intake resulting in hypoxia secondary to hypocapnia. In addition, respiratory alkalosis also causes cerebral vasoconstriction with concomitant cerebral hypoxia. Clinical treatment for respiratory alkalosis include supplemental oxygen or drug removal if the alkalosis is drug induced (Ayers and Warrington, 2008).

### Metabolic acidosis

Metabolic acidosis results from an increase in metabolic acid production or an inability to remove acid/reabsorb base in the kidneys. As a result there is a decrease in blood pH. Some of the causes of metabolic acidosis include diarrhea, severe renal failure, lactic acidosis, ketoacidosis, and drug intoxication. Clinical symptoms include a low blood pH (<7.35), chest pain, decreased cardiac output, hypotension, increased calcium release, and muscle weakness. Patients often display deep, labored breathing patterns (Kussmaul respiration) described as “air hunger.” Metabolic acidosis can lead to coma and death (Ayers and Warrington, 2008).

Similar to respiratory acidosis, the overall result of this insult is decreased blood pH and consequently increased ventilation rate. Metabolic acidosis causes upregulation of NHE3 (Kiwull-Schöne et al., 2007) and increase expression of NBCn1 in several brain regions (Cooper et al., 2009; Park et al., 2010). This predicted increase in *J_E_* would facilitate extra protection against intracellular and extracellular acid overload.

As an acute compensation, the body regulates the bicarbonate buffering system to drive the production of CO_2_ which can be eliminated through increased ventilation. The chemoreceptors in the brainstem and hypothalamus are activated and stimulate respiratory structures to increase breathing rate and elimination of CO_2_. The increased acid is also intrinsically buffered by proteins, phosphates, and carbonate in the bone. As a chronic compensation, the renal system increases the secretion of H^+^ (Giebisch and Windhager, 2009). Clinical treatment for metabolic acidosis includes normalizing blood volume and cardiac output. For more severe cases bicarbonate and acetate are administered and pCO_2_ is decreased (Ayers and Warrington, 2008).

### Metabolic alkalosis

Metabolic alkalosis results from an increase bicarbonate in the blood. This increase may be due to either a primary increase in bicarbonate reabsorption or be secondary to decreased production or increased loss of H^+^. As a result there is an increase in blood pH. Some of the causes of metabolic alkalosis include vomiting, diuretics, or increased urinary excretion of Cl^−^. Clinical symptoms include arteriolar constriction, reduced coronary blood flow, hypokalemia, tetany, and seizures (Ayers and Warrington, 2008).

The increased blood pH reduces the respiratory stimulus for breathing and hypoventilation occurs. As a result CO_2_ is retained and shuttled through the carbonic anhydrase buffering system and H^+^ is produced which lowers blood pH. A more chronic compensation occurs when the renal system decreases H^+^ secretion (Giebisch and Windhager, 2009). Clinical treatment for metabolic alkalosis includes volume replacement, use of carbonic anhydrase inhibitors (acetazolamide), and correction of potassium depletion (Ayers and Warrington, 2008). We are unaware of any reports of compensatory alterations in acid base transporter activity or expression in the brain under metabolic alkalosis.

## Summary

Neuronal excitability is highly susceptible to fluctuations in intra- and extracellular pH. It is the delicate balance between the actions of the acid-base transporters that contributes to *J*_L_ and *J*_E_, maintaining a permissive neuronal pH in the face of physiological and pathophysiological acid-base disturbances. Loss of any of these transporters is associated with profound neuronal abnormalities and conversely, disturbance in pH are associated with many different physiopathological conditions such as Alzheimer's and Parkinson's diseases. The contribution of acid-base transporters to the severity of the signs of neurological disorders is a promising area of investigation.

### Conflict of interest statement

The authors declare that the research was conducted in the absence of any commercial or financial relationships that could be construed as a potential conflict of interest.
